# Regulated Self-Folding in Multi-Layered Hydrogels Considered with an Interfacial Layer

**DOI:** 10.3390/gels10010048

**Published:** 2024-01-10

**Authors:** Jun Woo Lim, Sang Jin Kim, Jimin Jeong, Sung Gyu Shin, Chaewon Woo, Woonggyu Jung, Jae Hyun Jeong

**Affiliations:** 1 Department of Chemical Engineering, Soongsil University, Seoul 06978, Republic of Korea; ljw9424@soongsil.ac.kr (J.W.L.); sj1229v@soongsil.ac.kr (S.J.K.); jamiej1123@soongsil.ac.kr (J.J.); whitegd45@ssu.ac.kr (S.G.S.); chaewon311@soongsil.ac.kr (C.W.); 2 Department of Biomedical Engineering, Ulsan National Institute of Science and Technology (UNIST), Ulsan 44919, Republic of Korea

**Keywords:** multi-layered hydrogel, interfacial layer, self-folding, soft hydrogel actuators

## Abstract

Multi-layered hydrogels consisting of bi- or tri-layers with different swelling ratios are designed to soft hydrogel actuators by self-folding. The successful use of multi-layered hydrogels in this application greatly relies on the precise design and fabrication of the curvature of self-folding. In general, however, the self-folding often results in an undesired mismatch with the expecting value. To address this issue, this study introduces an interfacial layer formed between each layered hydrogel, and this layer is evaluated to enhance the design and fabrication precision. By considering the interfacial layer, which forms through diffusion, as an additional layer in the multi-layered hydrogel, the degree of mismatch in the self-folding is significantly reduced. Experimental results show that as the thickness of the interfacial layer increases, the multi-layered hydrogel exhibits a 3.5-fold increase in its radius of curvature during the self-folding. In addition, the diffusion layer is crucial for creating robust systems by preventing the separation of layers in the muti-layered hydrogel during actuation, thereby ensuring the integrity of the system in operation. This new strategy for designing multi-layered hydrogels including an interfacial layer would greatly serve to fabricate precise and robust soft hydrogel actuators.

## 1. Introduction

Multi-layered hydrogels consisting of bi- or tri-layers with different swelling ratios are designed to serve as soft hydrogel actuators through self-folding, which can be induced by external stimuli. These hydrogels have been extensively studied for their potential use as bio-robots [1], active actuators [2,3,4], and drug delivery systems [5,6], with a focus on adjusting their mechanical properties to achieve various shapes [7,8,9]. For instance, recent studies have explored their potential in developing innovative soft actuators designed for live cell stimulation through self-folding, showcasing the adaptability and precision in controlling their mechanical properties [2]. This soft actuator exhibits the capability to stimulate live cell clusters through a dynamic interplay of compression and tension, triggered by changes in temperature. The self-folding mechanism allows for the precise manipulation of the curvature, ensuring adaptability and precision in live cell stimulation.

**Figure 1:**
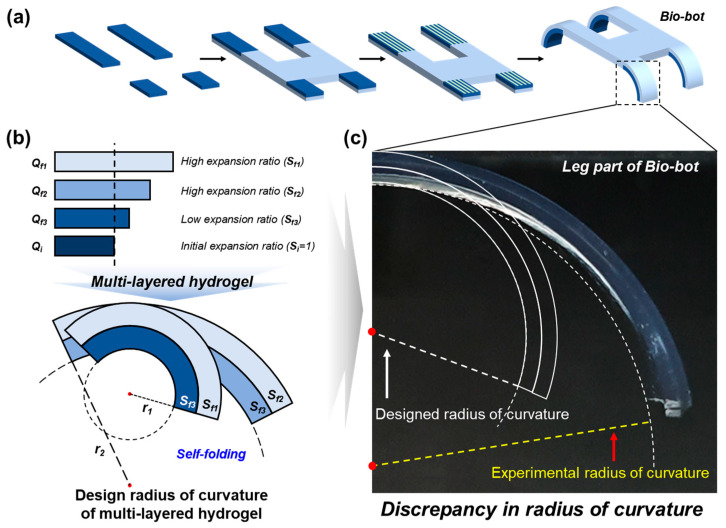
(**a**) Schematic description of the preparation of the biological machine (Bio-bot) by self-folding. (**b**) Schematic diagram illustrating the design of the radius of curvature for multi-layered hydrogels. The radius of curvature is predicted using the heat-induced bimetallic strip equation, with mechanical properties as the expansion ratio and elastic modulus of each layer. (**c**) Image depicting the radius of curvature of the multi-layered hydrogel, formed larger than the intended radius of curvature.

The successful utilization of multi-layered hydrogels in soft actuation critically depends on the precision with which the curvature of the self-folding is designed and fabricated [10,11,12]. Typically, the curvature of these multi-layered hydrogels has been predicted using the bimetallic strip equation, considering the mechanical properties of individual layers [13,14]. However, it is imperative to recognize the crucial role played by an overlooked component, the interfacial layer. The interfacial layer arises as a natural consequence of the manufacturing process, forming between adjacent layers of the multi-layered hydrogel through diffusion. Although not intentionally engineered, its presence exerts a substantial influence on the system’s behavior. Specifically, the self-folding process, which stands as a critical element in the functionality of these hydrogel actuators, is intricately influenced by the properties of this interfacial layer. Neglecting the interfacial layer when designing and calculating the curvature of self-folding, often using approaches like the bimetallic strip equation, can lead to discrepancies between the anticipated and actual outcomes (Figure 1) [15,16,17,18]. By considering this layer, formed through diffusion, in the bimetallic strip equation for a tri-layered hydrogel, we observe a significant reduction in the mismatch between predicted and experimental curvature values. The interfacial layer introduces a previously unaccounted factor that shapes the hydrogel’s response during self-folding.

In this study, we examine the significance of this interfacial layer, emphasizing that it should not be disregarded when considering multi-layered hydrogels. By incorporating this naturally occurring layer as an essential component of the system, a more accurate representation of the self-folding behavior can be achieved, aligning the theoretical results with practical observations. Furthermore, we demonstrate that this interfacial layer plays a crucial role in maintaining the structural integrity of the multi-layered hydrogel during actuation. Its presence prevents the separation of layers and contributes to the robustness of the entire system. It acts as a diffusion layer that crosslinks with both layers, effectively binding them together and ensuring that the two distinct layers do not separate during actuation, which is vital for the structural integrity of the multi-layered hydrogel system. Thus, the consideration of the interfacial layer is essential for precision in self-folding as well as the creation of robust and dependable soft hydrogel actuators. In conclusion, this study highlights the underestimated role of the interfacial layer in multi-layered hydrogels. By recognizing and incorporating this layer into the design and analysis, we bridge the gap between theory and reality, ultimately leading to more accurate, reliable, and resilient soft hydrogel actuators [19,20,21,22].

## 2. Results and Discussion

### 2.1. Mechanical Properties of Individual Layers in Multi-Layered Hydrogels

In this section, we present a comprehensive overview of the molecular structure and synthetic pathway employed to fabricate the bi-layered hydrogel. The choice of acrylamide (AAm) as the monomer and N,N′-methylenebisacrylamide (MBA) as the cross-linker, along with the incorporation of the initiator Irgacure2959, is detailed. The concentration variations and the rationale behind the selection of MBA concentrations in the first and second layers are discussed. Furthermore, we elaborate on the photo-crosslinking process and conditions, emphasizing their role in the formation of a well-defined bi-layered hydrogel.

**Figure 2:**
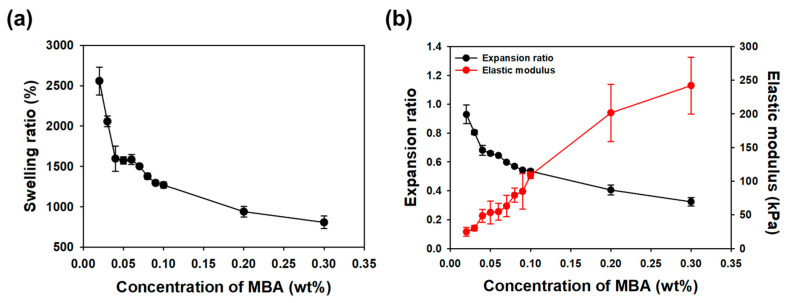
(**a**) The swelling ratio of the AAm hydrogels with varying MBA concentrations. (**b**) The expansion ratio and the elastic modulus of the AAm hydrogels at different MBA concentrations.

First, the investigation into the mechanical properties of hydrogels by varying the cross-linker concentration highlighted essential aspects of their behavior. The dependence of the expansion ratio (S) and elastic modulus (E) on the MBA concentration underscored the need to tailor these properties for specific applications, such as self-folding in multi-layered hydrogels. The expansion ratio and elastic modulus of the hydrogel, composed of AAm monomer at 20% (*w*/*v*) and varying concentrations of MBA (0.02–0.3% *w*/*v*), were measured. As the concentration of the cross-linker increased, the swelling ratio (S) and expansion ratio (E), calculated using the formulas described in the Materials and Methods section (Equations (1) and (2)), exhibited a decrease, while the elastic modulus (E) demonstrated an increase (see Figure 2 for details). The swelling ratio, calculated using Equation (1), represents the degree to which the hydrogel can absorb water depending on the mass (W_d_) of the total polymer comprising the hydrogel. However, when inducing self-folding in multi-layered hydrogels, it is more important to consider how much each layer of the hydrogel actually expands compared to its initial size. While conventional approaches focus on adjusting the swelling ratio, our study emphasizes the importance of considering the expansion ratio, particularly in the context of self-folding applications. Evaluating how each layer of the hydrogel expands compared to its initial size provides a more accurate prediction of the radius of curvature for multi-layered hydrogels. This challenges the traditional reliance on the swelling ratio alone. Moreover, the trends observed in Figure 2 reveal specific concentration-dependent behaviors, further supporting the argument for tailoring mechanical properties to achieve optimal self-folding characteristics in multi-layered hydrogels. In summary, our approach shifts the focus from the conventional emphasis on adjusting the swelling ratio to a more nuanced consideration of the expansion ratio in multi-layered hydrogels. By evaluating how each layer expands relative to its initial size, we gain a more precise understanding of self-folding dynamics.

### 2.2. Design and Fabrication of Multi-Layered Hydrogels

**Figure 3:**
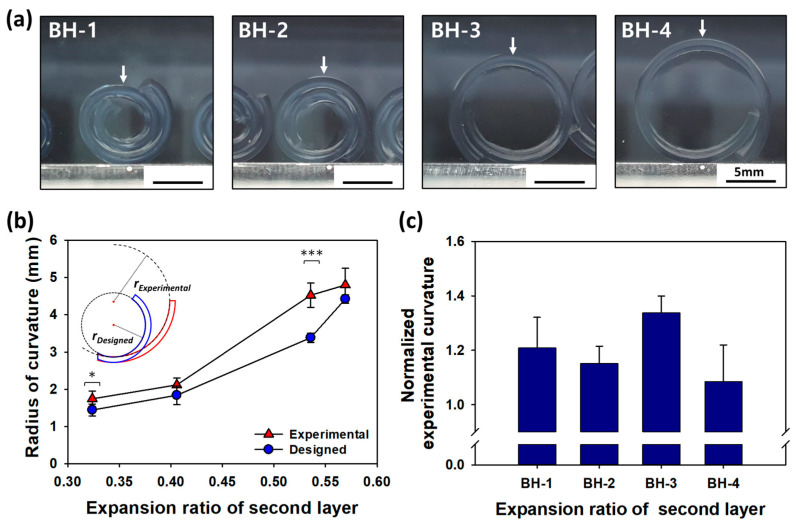
(**a**) Bright field images of bi-layered hydrogels with varying expansion ratios of the second layer. The abbreviations BH-1, BH-2, BH-3, and BH-4 correspond to different bi-layered hydrogel samples. (**b**) Calculated radius of curvature for the bi-layered hydrogel using a mathematical model developed for the curvature of a heat-induced bimetallic strip. Comparison of designed and experimental radius of curvatures based on differences in the expansion ratio of the two layers. (* *p* < 0.05, *** *p* < 0.01) (**c**) Normalized experimental curvature of BH-1, 2, 3, 4. Experimental curvatures for BH-1, 2, 3, 4 have been normalized to the designed radius of curvature.

A bi-layered hydrogel was formed using a photo-crosslinking process, consisting of two layers. The first layer was crosslinked using a fixed concentration of MBA (0.04% *w*/*v*), while the concentration of the second layer was adjusted to 0.08–0.3% (*w*/*v*). The radius of curvature (r) was calculated using the formulas described in the Materials and Methods section (Equation (4)) to predict the curvature of the multi-layered hydrogel upon self-folding. The calculated radius of curvature was then compared to the radius of curvature of the fully expanded bi-layered hydrogel (Figure 3a). However, the experimental radius of curvature of the bi-layered hydrogel did not match the calculated radius of curvature. For example, in the case of Bi-layered hydrogel (BH), such as BH-1, which had a difference in the expansion ratio of 0.11 between the two layers, it was predicted to be 1.44 mm. However, the experimental radius of curvature was 1.74 mm, representing a 1.2-fold difference from the designed radius of curvature. The curvature of BH-3 was also found to be significantly different from the designed radius of curvature, with an approximately 1.3-fold difference (see Figure 3b,c for details). These results indicate a significant discrepancy between the design value and the experimental value when predicting the radius of curvature using the bi-layered equation. Despite the observed disparities between the calculated and experimental values for the radius of curvature in the bi-layered hydrogel, our study emphasizes the significance of these findings in refining predictive models and underscores the need for a more comprehensive understanding of the underlying factors influencing self-folding behavior in multi-layered hydrogel systems.

### 2.3. Curvature Simulation of Multi-Layered Hydrogels

**Figure 4:**
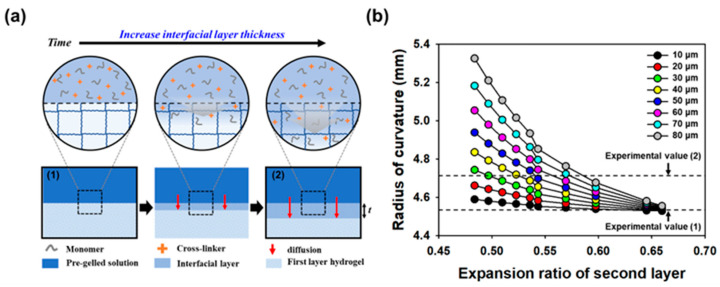
(**a**) Schematic representation of the formation of the interfacial layer through the diffusion of monomers and cross-linkers. (**a-1**) Designed curvature in the absence of the interfacial layer formation, (**a-2**) Experimental curvature observed after the interfacial layer formation. (**b**) Simulation of the radius of curvature was conducted by varying the expansion ratio and the thickness of the interfacial layer in the hydrogel. The arrow marks the experimental radius of curvature of the bi-layered hydrogel, which was prepared with the composition of BH-3.

The experimental radius of curvature of the bi-layered hydrogel was observed to deviate significantly from the originally designed radius of curvature. This discrepancy was attributed to the formation of an interfacial layer at the interface between the two layers due to diffusion during the fabrication of the second layer of the multi-layered hydrogel (see Figure 4a for details). To examine this phenomenon in more detail, simulations were conducted, assuming that the physical properties of the interfacial layer fell between those of the first and second layers. Given that the interfacial layer naturally formed during the preparation of the multi-layered hydrogel, the bi-layered hydrogel was effectively treated as a tri-layered hydrogel for the purpose of simulations. The radius of curvature of the tri-layered hydrogel was then predicted using Equation (1) to account for the presence of the interfacial layer, employing MATLAB for the simulation.

(1) $$r=\frac{2\left[\frac{∆\varepsilon_{12}\left(t_{1}+t_{2}\right)}{t_{3}E_{3}}+\frac{∆\varepsilon_{23}\left(t_{2}+t_{3}\right)}{t_{1}E_{1}}+\frac{∆\varepsilon_{13}\left(t_{1}+2t_{2}+t_{3}\right)}{t_{2}E_{2}}\right]}{\frac{{\left.{\left(t\right.}_{1}+t_{2}\right)}^{2}}{t_{3}E_{3}}+\frac{{\left.{\left(t\right.}_{2}+t_{3}\right)}^{2}}{t_{1}E_{1}}+\frac{{\left.{\left(t\right.}_{1}+2t_{2}+t_{3}\right)}^{2}}{t_{2}E_{2}}+\frac{4(\sum_{1}^{3}E_{n}\frac{{t_{n}}^{3}}{12})(\sum_{1}^{3}\left.{t_{n}E}_{n}\right)}{t_{1}E_{1}t_{2}E_{2}t_{3}E_{3}}}$$

To corroborate the impact of the interfacial layer on the radius of curvature of the multi-layered hydrogel, BH-3 and BH-5 were prepared by altering the order of layer preparation. The resulting radius of curvature is displayed as Experimental values (1) and (2) in Figure 4b. The composition of the tri-layer hydrogel mirrored that of BH-3, but the thickness of the interfacial layer was varied from 10 to 80 μm, while the expansion ratio ranged from 0.484 to 0.660. The arrow in Figure 4b represents the experimental radius of curvature. These findings demonstrate that the radius of curvature of the bi-layered hydrogel can be precisely designed and predicted by taking into account the thickness of the interfacial layer. Furthermore, this meticulous exploration into the interfacial layer’s impact on the radius of curvature provides valuable insights for enhancing the predictability and precision of multi-layered hydrogel designs. The comprehensive analysis sheds light on the interplay of the layer formation order, thickness variations, and expansion ratios, enabling more informed and tailored fabrication of hydrogels for diverse applications.

In particular, the experimental value (1) of BH-3, prepared with the first layer having an MBA concentration of 0.04% (*w*/*v*), measured at 4.53 mm. This measurement matched the design value of the curvature radius of the tri-layered hydrogel, complete with a 10 μm interfacial layer and an expansion ratio of 0.660. Conversely, the experimental value (2) of BH-5, formed with the first layer having an MBA concentration of 0.08% (*w*/*v*), recorded a radius of curvature of 4.71 mm. The radius of curvature of the multi-layered hydrogel with experimental value (2) was estimated to have an interfacial layer thickness exceeding 30 μm. However, the interfacial layer, formed through diffusion, cannot expand beyond the first layer, with an expansion ratio lower than 0.536. Hence, it is reasonable to infer that the interfacial layer had a thickness ranging from 30 and 60 μm. These results affirm that the experimental and designed radius of curvature align when employing the radius of curvature equation for tri-layered hydrogels, which considers the interfacial layer as one layer in the bi-layered hydrogels. These findings highlight the nuanced impact of the MBA concentration on the experimental outcomes and emphasize the importance of accounting for interfacial layer dynamics in accurately predicting the radius of curvature in multi-layered hydrogel systems.

### 2.4. Evaluation of the Interfacial Layer in Multi-Layered Hydrogels

**Figure 5:**
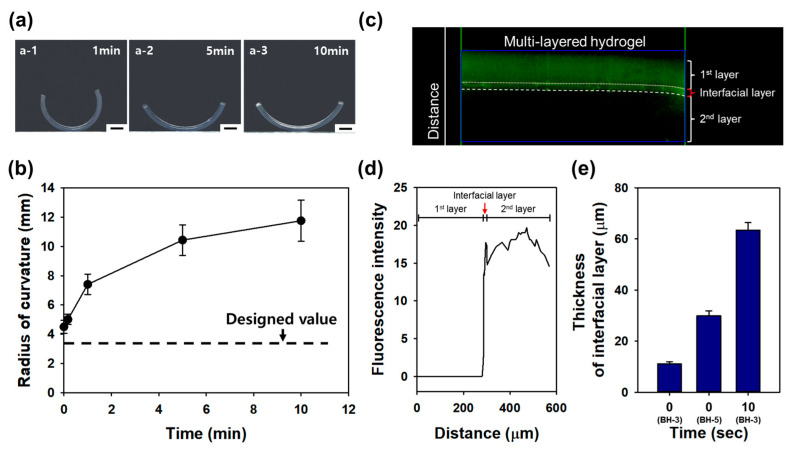
(**a**) Bright field images of bi-layered hydrogels with varying diffusion times for the second layer ((**a-1**): 1 min; (**a-2**): 5 min; (**a-3**): 10 min). (**b**) Radius of curvature of bi-layered hydrogels relative to the diffusion time of the second layer. (**c**) Cross-sectional fluorescence image of a multi-layered hydrogel with the first and second layers, including the interfacial layer. (**d**) Fluorescence intensity across the multi-layered hydrogel as a function of distance. (**e**) Thickness measurements of the interfacial layer in each bi-layered hydrogel (BH-3 and BH-5) at 0 and 10 s. (Scale bar: 2 mm).

To investigate the impact of the interfacial layer’s thickness on the radius of curvature of bi-layered hydrogels, the diffusion time was adjusted to various intervals, including 0, 0.2, 1, 5, and 10 min. Bi-layered hydrogels with BH-3 composition were created using a fixed concentration of 0.04% (*w*/*v*) MBA at the first layer. As shown in Figure 5(a-1–a-3), it becomes visually evident that the radius of curvature increases with longer diffusion times. The bi-layered hydrogel fabricated without any diffusion time and the one with 10 min of diffusion time exhibited 1.3-fold and 3.5-fold increases from the designed radius of curvature, respectively (Figure 5b). These results underscore the crucial role of the diffusion time in determining the interfacial layer’s thickness, directly influencing the radius of curvature in bi-layered hydrogels. This insight offers a systematic approach to tailoring hydrogel designs for specific applications by controlling diffusion parameters during fabrication.

Notably, the experimental radius of curvature in the bi-layered hydrogel with 10 s of diffusion time measured 5.01 mm, indicating that the thickness of the interfacial layer had grown to more than 60 μm, as simulated. These findings underscore the importance of the rapid manufacturing of multi-layered hydrogels to align with the designed radius curvature. Furthermore, to verify the actual thickness of the interfacial layer, a bi-layered hydrogel was prepared by introducing 0.0005% (*w*/*v*) of Fluorescein O,O′-dimethacrylate (Sigma, St. Louis, MO, USA) into the second layer. Immediately after fabrication, an image was captured using a fluorescence microscope (Figure 5c), and the thickness of the interfacial layer and each layer was measured based on the fluorescence intensity and distance within the image (Figure 5d). Bi-layered hydrogels were prepared with diffusion times of 0 and 10 s for BH-3 and 0 s for BH-5. As a result, the thickness of the interfacial layer in BH-3 and BH-5 at 0 s was measured as 11.10 ± 0.82 μm and 29.87 ± 1.92 μm, respectively. Additionally, the thickness of the interfacial layer of BH-3 at 10 s was measured as 63.42 ± 2.91 μm (Figure 5e). These results underscore the importance of considering the interfacial layer in the design of multi-layered hydrogel curvatures, aligning with previous simulation findings. In light of our findings on the interfacial layer’s growth under varying diffusion times, we anticipate the need to precisely control the formation of interfacial layers and measure their properties for more accurate regulation.

**Figure 6:**
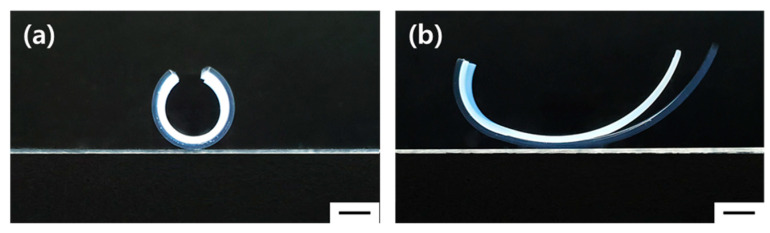
Bright field images of bi-layered hydrogels composed of PEGDA575 for the first layer and PEGDA3400 for the second layer, (**a**) with proper diffusion curves at the interface, and (**b**) with a minimal interfacial layer. (Scale bar: 2 mm).

On a different note, to assess the impact of diffusion of structural robustness, a multi-layered hydrogel was prepared using PEGDA. A bi-layered hydrogel was crafted using PEGDA575 at 20% (*w*/*v*) for the first layer and PEGDA3400 at 20% (w/v) for the second layer. Upon separating the prepared PEGDA bi-layered hydrogel into two layers, it was observed that the two layers separated due to inadequate diffusion at the interface (Figure 6b). This result corroborates our findings that the structural integrity of the multi-layered hydrogel is maintained only when proper diffusion occurs at the interface (Figure 6a). The interfacial layer acts as a diffusion layer that crosslinks with both layers, effectively binding them together and ensuring that the two distinct layers do not separate during actuation, which is vital for the structural integrity of the multi-layered hydrogel system.

Therefore, it is essential to consider diffusion at the interface of each layer during the design and fabrication of multi-layered hydrogels. The interfacial layer formed by diffusion must also be taken into account. This approach minimizes experimental errors by enabling accurate design and facilitating the prediction of the mechanical properties of the interfacial layer generated through diffusion. Furthermore, the diffusion layer is critical for ensuring the robustness of the system and preventing layer separation during actuation. In summary, the design of multi-layered hydrogels that accounts for the formation of the interfacial layer due to diffusion holds vast applications in fields such as bio-robotics, actuators, tissue engineering, and drug delivery [23,24,25].

## 3. Conclusions

In this study, we introduced an innovative strategy to enhance the precision and robustness of multi-layered hydrogels as soft hydrogel actuators by focusing on the interfacial layer, a natural byproduct of the manufacturing process formed by diffusion between the layers. Our experiments unequivocally reveal that as the interfacial layer’s thickness increases, self-folding becomes significantly more predictable and precise, leading to a remarkable 3.5-fold increase in the radius of curvature. We also unveiled the interfacial layer’s pivotal role in preserving structural integrity during the actuation. This groundbreaking approach not only reconciles theory with practice but also promises the development of highly accurate and reliable soft hydrogel actuators. The deliberate incorporation of the interfacial layer into the multi-layered hydrogel design represents a significant advancement with transformative potential in bio-robots, active actuators, tissue engineering, and drug delivery applications.

## 4. Materials and Methods

### 4.1. Hydrogel Preparation

Hydrogels were prepared using acrylamide (acrylamide, AAm, Sigma) and methylenebisacrylamide (N,N′-methylenebisacrylamide, MBA, Sigma) as monomers. The total concentration of AAm was fixed at 20% (*w*/*v*) and prepared by adjusting the concentration of MBA between 0.02% to 0.3% (*w*/*v*). An initiator, Irgacure2959 (2-hydroxy-40-(2-hydroxyethoxy)-2-methylpropiophenone, Sigma), was added to reach a final concentration of 0.2% (*w*/*v*). The hydrogels were formed by exposure to ultraviolet (UV) light (365 nm, VILBER LOURMAT, 4W) for 10 min. Subsequently, the gels were punched into 8 mm diameter disks and incubated in deionized (DI) water until fully swollen before characterization. The resulting hydrogels demonstrated tunable properties, showcasing the versatility of this synthesis approach for creating hydrogels with a range of mechanical and swelling characteristics suitable for diverse applications.

### 4.2. Characterizations of Hydrogels

The weight of hydrogel (*W_s_*) was measured after 12 h of incubation in DI water. The weight of the dried hydrogel (*W_d_*) was determined after drying at 60 °C over 24 h. The swelling ratio (*Q_m_*) was calculated using Equation (2).

(2) $$Q_{m}=\left(\frac{W_{s}-W_{d}}{W_{d}}\right)\times 100$$

The expansion ratio, crucial for inducing self-folding in multi-layered hydrogels with equal dimensions for each layer, was characterized using the swelling ratio of hydrogels when prepared (*Q_i_*) and after full incubation in DI water (*Q_f_*). The expansion ratio, indicating one-dimensional expansion of the hydrogel, was calculated using Equation (3).

(3) $$S={\left(\frac{Q_{f}}{Q_{i}}\right)}^{\frac{1}{3}}-1$$

Here, *Q_i_* and *Q_f_* represent the swelling ratio (*Q_m_*) before and after immersion in the aqueous solution. The mechanical properties of the hydrogel were evaluated using a universal testing machine (UTM, DrTech, Seongnam-si, Korea). Hydrogels, standardized to 8 mm diameter and 1 mm height, were compressed at a 10% strain and a constant rate of 1.0 mm/min, with a load range of 1.0 kg·f after incubation over 24 h.

### 4.3. Design and Fabrication of the Multi-Layered Hydrogels

The radius of curvature (*r*) of multi-layered hydrogels was determined using the bimetallic strip curvature Equation (4) [9,12]. In Equation (4), *E*_1_ and *E*_2_ represent the elastic modulus of each layer, *t*_1_ and *t*_2_ represent the thickness of each layer, and ∆*ε* represents the difference of expansion ratio of each layer.

(4) $$r=\frac{{E_{1}}^{2}{t_{1}}^{4}+4E_{1}E_{2}{t_{1}}^{3}t_{2}+6E_{1}E_{2}{t_{1}}^{2}{t_{2}}^{2}+4E_{1}E_{2}t_{1}{t_{2}}^{3}+{E_{2}}^{2}{t_{2}}^{4}}{6E_{1}E_{2}(t_{1}+t_{2})t_{1}t_{2}∆\varepsilon }$$

The multi-layered hydrogels were composed of bi-layered hydrogels with different compositions (Table 1). The concentration of each layer of the bi-layered hydrogel was fixed at a final concentration of 20% (*w*/*v*) for AAm and 0.2% (*w*/*v*) for Irgacure2959. The MBA concentration in the first layer was fixed at 0.04% (*w*/*v*), while the second layer’s concentration was adjusted to 0.08–0.3% (*w*/*v*) to vary the radius of curvature. The bi-layered hydrogel was assembled by first creating a hydrogel through UV exposure, followed by the preparation of second hydrogel layer on top of the first. The resulting multi-layered hydrogels were formed as strips measuring 30 mm in length and 5 mm in width, and 3 mm in thickness for each layer. The radius of curvature of the multi-layered hydrogels was measured after swelling in DI water for 24 h. The systematic control of the hydrogel composition and layer-specific adjustments allowed for the precise modulation of mechanical properties, enabling the tailoring of multi-layered hydrogels for applications demanding specific curvature characteristics and diverse functionalities.

### 4.4. Characterization and Simulation of Multi-Layered Hydrogels

The disparity between the designed and experimental radius of curvature (*r*) concerning the thickness of the interfacial layer was investigated through simulation. The radius of curvature was simulated using Equation (4) to calculate the radius of curvature of the tri-layered hydrogel, considering the presence of the interfacial layer in the bi-layered hydrogel. The thickness of the first and second layers of the bi-layered hydrogel was set at 300 μm for each, and the thickness of the interfacial layer was varied within the range of the first layer. The thickness of the interfacial layer was adjusted within the range of 10–60 um, and the composition of the first and second layers was the same as that of BH-3 in Table 1. The physical properties of the interfacial layer were determined within the mechanical properties range of the two layers. The simulations not only revealed insights into the correlation between the interfacial layer thickness and the observed disparity but also provided a basis for refining the design parameters for achieving precise control over the curvature in multi-layered hydrogel systems.

In addition, the diffusion time was adjusted to 0, 0.2, 1, 5 and 10 min to prepare a bi-layered hydrogel to determine the effect of the interfacial layer in multi-layered hydrogels. The multi-layered hydrogel was prepared in the same manner as in sample 3. Additionally, a multi-layered hydrogel was prepared using poly(ethyleneglycol) diacrylate (PEGDA) to confirm the effect of diffusion based on molecular weight. The concentration of the first layer was fixed at 20% (*w*/*v*) with PEGDA575 (Mn of 575 g/mol, Sigma), and concentration of the second layer was fixed at 20% (*w*/*v*) with PEGDA3400 (Mn of 3400 g/mol, Alfa Aesar). Irgacure2959 was added to reach a final concentration of 0.2% *w*/*v*) as an initiator. The variation in diffusion times and the utilization of poly(ethyleneglycol) di-acrylate (PEGDA) in multi-layered hydrogel synthesis further contribute to our understanding of interfacial layer dynamics, offering valuable insights for optimizing the design and performance of such hydrogel systems.

## Figures and Tables

**Table 1:** Preparation of bi-layered hydrogels (BH) with varying expansion ratio of second layer.

Sample	First Layer				Second Layer			
	Aam ^a^	MBA ^b^	Irgacure 2595 ^c^	Expansion Ratio ^d^	Aam ^a^	MBA ^b^	Irgacure 2595 ^c^	Expansion Ratio ^d^
BH-1	20	0.04	0.2	0.681	20	0.30	0.2	0.569
BH-2	20	0.04	0.2	0.681	20	0.20	0.2	0.536
BH-3	20	0.04	0.2	0.681	20	0.10	0.2	0.406
BH-4	20	0.04	0.2	0.681	20	0.08	0.2	0.385
BH-5	20	0.04	0.2	0.406	20	0.04	0.2	0.681

^a^ Concentration of monomer %(*w*/*v*), ^b^ Concentration of cross-linker %(*w*/*v*), ^c^ Concentration of photo-initiator %(*w*/*v*), ^d^ Expansion ratio of each layer.

## Data Availability

All data and materials are available on request from the corresponding author. The data are not publicly available due to ongoing researches using a part of the data.
